# Risk Factors for Chronic Diseases and Multimorbidity in a Primary Care Context of Central Argentina: A Web-Based Interactive and Cross-Sectional Study

**DOI:** 10.3390/ijerph14030251

**Published:** 2017-03-02

**Authors:** David E. V. Olivares, Frank R. V. Chambi, Evelyn M. M. Chañi, Winston J. Craig, Sandaly O. S. Pacheco, Fabio J. Pacheco

**Affiliations:** 1 Center for Health Sciences Research, School of Medicine & Health Sciences, Universidad Adventista del Plata, Libertador San Martín, 25 de Mayo 99, Entre Ríos 3103, Argentina; d.e.vera@hotmail.com (D.E.V.O.); frankvargasch@gmail.com (F.R.V.C.); evymar80@gmail.com (E.M.M.C.); sandalyoliveira@doc.uap.edu.ar (S.O.S.P.); 2 Institute for Food Science and Nutrition, Universidad Adventista del Plata, Libertador San Martín, 25 de Mayo 99, Entre Ríos 3103, Argentina; wcraig@andrews.edu; 3 Department of Public Health, Nutrition and Wellness, School of Health Professions, Andrews University, Berrien Springs, MI 49104, USA

**Keywords:** chronic diseases, risk factors, multimorbidity, primary health care, sociodemographic, lifestyle habits, anthropometrics, blood test analysis, community-based primary care, Argentina

## Abstract

Global health agencies estimate an increase of chronic diseases in South America. Nevertheless, few studies have investigated chronic diseases and their risk factors in the perspective of multimorbidity. This research aimed to identify these aspects in a primary health care setting of central Argentina. The Pan America version of the STEP wise approach surveillance (STEPS) instrument of the World Health Organization was applied to 1044 participants, 365 men and 679 women, with a mean age of 43 years. High prevalence of overweight (33.5%), obesity (35.2%), central obesity (54%), dyslipidemia (43.5%), metabolic syndrome (21.1%), low intake of fruit and vegetables (91.8%), low levels of physical activity (71.5%), risky alcohol consumption (28%), and smoking (22.5%) were detected. Hypertension and diabetes were the most prevalent chronic conditions and the total prevalence of multimorbidity was 33.1%, with 2, 3, 4, 5 and 6 chronic conditions found in 19.9%, 9.1%, 2.6%, 1.1% and 0.4% of the population, respectively. Multimorbidity affected 6.4% of the young, 31.7% of the adults, and 60.6% of the elderly, and was more prevalent among women, and in participants with lower levels of education. Having multimorbidity was significantly associated with obesity, central obesity, and higher concentrations of total blood cholesterol, low-density lipoprotein cholesterol, triglycerides, and glucose. A website was made available to the participants in order to share the experimental results and health-promoting information.

## 1. Introduction

According to a report from the World Health Organization (WHO), chronic diseases kill 38 million people worldwide each year [1]. It is estimated that by the year 2030 around 52 million people worldwide will die from chronic diseases [2]. Cardiovascular diseases (CVD) account for the majority of deaths, 17.5 million each year, followed by cancer, 8.2 million, respiratory diseases, 4 million, and diabetes 1.5 million [1,2]. Nearly 75% of CVD deaths occur in low- and middle-income countries, affecting men and women equally [1,2]. In South America, chronic diseases cause approximately 77% of deaths, and in Argentina, chronic diseases account for 81% of the total number of deaths [3,4,5].

A risk factor is any trait, characteristic or exposure of an individual that increases their likelihood of suffering from a disease or injury [6]. These can be non-modifiable or modifiable, major or conditioning [1]. Early identification and proper management of risk factors associated with chronic diseases is essential for developing appropriate health policies to intervene at different levels, especially at the primary health care (PHC) level. In Argentina, some modifiable risk factors have been strongly associated with chronic morbidity and higher mortality and include tobacco smoking, risky alcohol consumption, unhealthy dietary patterns, low level of physical activity (PA), high blood pressure (BP), and overweight/obesity [7].

However, not all risk factors are present in populations alike [2,8]. This is of importance since investigating individual risk factors for specific populations is fundamental to develop effective preventive strategies. In turn, the variation in time of these risk factors is an important consideration in surveillance for improving health interventions. Studies conducted in different parts of the world have shown that community-based primary care interventions are effective in reducing risk factors for chronic diseases [9,10,11]. eHealth is a fast-growing form of healthcare practice supported by electronic processes for reaching communities and facilitating interaction with health professionals. eHealth strategies have been successfully used in health interventions in the PHC setting, optimizing preventive care [12]. A recent study found that a web-based personalized approach to manage risk factors for CVD proved feasible and effective to reduce multiple unhealthy behaviors [13].

Considering that a highly prevalent chronic health condition, such as CVD and diabetes, may share a number of similar risk factors, it is not unusual to find more than one chronic disease in a single individual. Multimorbidity, a complex long-term health problem, commonly defined as the presence of two or more chronic medical conditions in the same individual [14,15], has been associated with reduced physical and mental functioning [15,16], decreased quality of life [17,18], absence due to sickness [19], increased health-associated complications and hospitalizations [20], polypharmacy [21], and great health expenditures [22]. There are many factors involved with multimorbidity, but socioeconomic and demographic factors, together with lifestyle habits are considered important determinants associated with multimorbidity [23,24,25,26,27]. Nevertheless, national as well as regional variances in epidemiological characterizations of these determinants for multimorbidity have been reported recently [28]. Hence, investigating specific epidemiological characteristics of multimorbidity, inter- and intra-population variations of its determinants, is necessary for effective public health management of this condition.

Considering the health burden of chronic diseases for South America and the Caribbean, new research on multimorbidity in these areas of the world is warranted [29]. Although studies have shown that proper epidemiological characterization of multimorbidity is of great importance, it has been little explored, with few references in the literature for this region of the world. In addition, the number of intervention studies targeting multiple risk factors for chronic diseases accounts for only 0.5% of all research being done in South America [30]. Therefore, this study aimed to identify the risk factors for chronic diseases connected with the health-related lifestyle habits, anthropometrics and blood biochemical profile, and the characteristics associated with multimorbidity in a population of the central area of Argentina. We have also looked at whether a website designed for this project, and used in a community primary care setting, would be useful for communicating specific health-promoting information for this population.

## 2. Materials and Methods

### 2.1. Study Participants and Setting

This is a cross-sectional analysis of the baseline data from a health promotion activity program developed in a socioeconomically disadvantage area of central Argentina by the River Plate Adventist University School of Medicine in conjunction with the local-regional PHC system. This project was conducted in the city of Diamante, between the years 2014 and 2015, province of Entre Ríos, Mesopotamia and central area of Argentina, located in a semi-urban zone. 

The total population of Diamante in the year 2010 was 19,930 inhabitants, 9525 men and 10,405 women [31]. People 18 years of age or older attending the PHC system or contacted during community health visits were randomly selected and invited to participate in the study. A total of 1044 persons, including 365 men and 679 women, were enrolled in the study.

At the beginning of the project, each participant received an oral invitation followed by a written description of the main steps of the study. Participants signed the written informed consent. All procedures associated with this project were conducted following the international ethical standards proposed by the Helsinki protocol for human research and this study was reviewed and approved by the Research Review Board (registered under the #44.2011) and Ethics Committee of the River Plate Adventist University School of Medicine (resolution #03-01-02/2012/2-2012).

### 2.2. Data Collection

Participants were contacted in person at least three different times during community visits and/or PHC clinic visits. Data were collected during these visits. Firstly, standard sociodemographic, lifestyle and health-related questionnaires were provided. At a second visit, anthropometric measurements were taken and completed questionnaires were collected. At the third scheduled visit, following a telephone call, a blood sample was obtained. Additional contacts were made to share results with participants.

In order to optimize data collection and follow-up, the city map of Diamante was strategically divided into 24 zones, according to the distribution of residencies, places of work, and the location of the public PHC areas of services (areas of reference for community health attention with sporadic medical attendances), and PHC clinic (community health attention with general practice physicians and nurses, and other PHC professionals). Due to the lack of an updated map of the city of Diamante, a project map had to be created based on field visits combined with information from previous maps of the city. The project map was generated using the Adobe Photoshop Extended CS 5 program (San Jose, CA, USA). The Supplementary Figures S1 and S2 shows the Diamante city and the project map of the city, respectively, containing the 24 zones and the distribution of participants, with their individual code number, spread in different areas of the city of Diamante.

The Pan America version of the WHO STEP wise approach surveillance (STEPS) instrument for measurements of non-communicable disease (NCD) risk factors was used in this study, adjusting some question of the generic questionnaire to the characteristics of this study [32]. The main sections of the instrument included sociodemographic and lifestyle information, tobacco use, alcohol consumption (AUDIT-C), history of chronic diseases, anthropometric measurements, and blood biochemical analysis for CVD and metabolic risk factors.

Briefly, participants provided information on age, gender, education, employment status, contact address, and telephone number. Data also collected included, cigarette smoking, alcohol consumption (risky alcohol consumption according to the AUDIT-C instrument, score of ≥4 points for men and ≥3 points for women) [33,34], physical activity (30 min or more of moderate aerobic activity at least three or more times/week on a regular basis), and the habitual daily intake of five or more portions of fresh fruit and vegetables. The history of arterial hypertension, CVC diseases, dyslipidemia, diabetes, cancer, chronic respiratory diseases, thyroid dysfunctions, celiac disease, rheumatoid arthritis, depressive disorders, and other chronic health conditions were assessed.

Blood pressure (BP) measurements were obtained with the participant in a seated position with a standard aneroid sphygmomanometer placed in the left arm using the BP cuff size 11 or 12, depending on the adult size of the arm, at the heart level. BP was measured twice during examination and averages were calculated. Participants were weighed in an orthostatic position without shoes, in light clothing, on an electronic scale. Body height was obtained by measuring to the nearest 0.1 cm with a non-extendable tape, without shoes in orthostatic position, except one participant seated in wheelchair. Waist circumference was also measured in orthostatic position, at the midpoint between the last rib and the iliac crest, with a flexible measuring tape to the nearest 0.1 cm. Body mass index (BMI) was calculated from weight (kg)/height (m^2^).

The recommendations of the Joint National Committee on Prevention, Detection, Evaluation and Treatment of High Blood Pressure 7 [35] were considered and high BP was defined as mean systolic blood pressure (SBP) ≥140 mmHg and/or mean diastolic blood pressure (DBP) ≥90 mmHg. History of medical diagnosis of hypertension, current use of antihypertensive medication and previous BP examination were registered. Central obesity was defined as ≥102 cm in men and ≥88 cm in women [36].

A 5 mL venous blood sample was withdrawn from the left or right arm after an overnight fast of approximately 12 h. The blood specimen was centrifuged within 45 min of the drawing at 5000 rpm for 10 min. The blood serum was stored at −18 °C for blood analysis of glucose, triglycerides, total cholesterol, high-density lipoprotein cholesterol (HDL-C) and low-density lipoprotein cholesterol (LDL-C). The samples (packed in ice) were transferred to the laboratory of the university-affiliated hospital of the School of Medicine within 20 days of the blood drawing and measurements were performed with the ARCHITECT c4000 clinical chemistry analyzer (Abbott Diagnostics). The following methods were used, Hexokinase-UV for glucose; CHOD-PAP for cholesterol; GPO-PAP for triglycerides; and direct method for HDL-C and LDL-C (Abbott Diagnostics reagents). LDL was measured in the blood serum specimen with triglycerides ≥200 mg/dL, for lower levels, triglycerides was indirectly determined using the Friedewald equation, LDL = total cholesterol-HDL- triglycerides/5 (mg/dL) [37].

Dyslipidemia was defined according to The Third Report of the NCEP Expert Panel on Detection, Evaluation, and Treatment of High Blood Cholesterol in Adults recommendations as total cholesterol of ≥200 mg/dL, LDL-C ≥130 mg/dL, triglycerides ≥150 mg/dL and HDL-C <40 mg/dL, and previous medical diagnosis or use of current medication for dyslipidemia [38]. A medical history of diabetes and the current use of antidiabetic medications were registered and altered fasting blood glucose concentration ≥110 mg/dL were defined according to the American Diabetes Association [39].

Metabolic syndrome was defined with the presence of at least three conditions: fasting blood glucose ≥110 mg/dL, abdominal obesity ≥102 cm for men and ≥88 cm for women, blood pressure ≥130/85 mmHg or being on antihypertensives, triglycerides ≥150 mg/dL or on treatment with lipid lowering agents, HDL-C <40 mg/dL men or <50 mg/dL in women [38,40].

The combined prevalence of several risk factors for chronic diseases was assessed in participants and included those aspects associated with obesity, central obesity, smoking, risky alcohol consumption, low intake of fruit and vegetables, low level of PA, and altered blood levels of LDL-C, HDL-C, triglycerides, and glucose.

### 2.3. Web Page Design and Devolution of Results

A website (www.diamantesalud.com) was generated for providing information about the project and for interaction with participants. The website was designed using HTML5 (Hypertext Markup Language) and contained four sections including general information of the study, health information, contact information and a search bar, which enabled participants to enter their project individual ID number and have access to the blood test analysis, anthropometric measurements and lifestyle information.

The website was connected to a “Google Analytics” account to determine the number of sessions (period in which a user interacts with the website, application, etc.), new users (number of first-time users during a selected period of time), number of pages visited, pages per session (average viewed pages and average duration of a session). The data was filtered by date from 1 December 2014 to 30 June 2015, and then by country (Argentina), thus eliminating incorrect measurements given out by computer robots and spiders. Filters applied also selected the time of interaction with the webpage, of at least one minute, eliminating unintentional and random webpage visitors. All images, graphics and vectors were used with previous permission or were under a “Creative Commons 0” license.

Participants, who had no access to the Web, were invited to use the community computer center located in the same building of the main PHC clinic, located in zone 1 of the city map, to have Web access viewing information associated with the project. In case subjects were not able to visit the community computer center for internet access, a printed version of the results was delivered personally to each participant of the study.

### 2.4. Statistical Analysis

The sample size estimated for the population of Diamante was of 1013 individuals, considering a 95% confidence level and a 3% precision. Descriptive analyses were carried out for sociodemographic, health information and risk factors for chronic diseases. Chi-square test was used for assessing differences in categorical variables between groups. To compare anthropometrics, lifestyle factors and biochemical values between groups, we used the *t* test and Mann–Whitney U test according the normality of variables. SPSS Inc., (Chicago, IL, USA) version 17 software was used for statistical analyses. *p* values < 0.05 were considered statistically significant.

## 3. Results

### 3.1. Population Characteristics

The main sociodemographic characteristics of participants and the self-reported medical history of chronic diseases as well as the prevalence of multimorbidities for this population are presented in Table 1. Subjects had a mean age of 43 years old (±15), were predominantly women, with an education level of primary school, were actively working and married. Hypertension, diabetes and dyslipidemia were the chronic health conditions most prevalent among the NCDs reported by the participants. The total prevalence of multimorbidity for this population was 33.1%, and the co-occurrence of multiple chronic health conditions is shown in Table 1.

### 3.2. Anthropometry, Blood Pressure and Blood Test Analysis

Table 2 shows the anthropometrics, blood pressures, and the blood lipids and blood glucose of participants. The mean general BMI of participants was of 28 ± 6.65 (kg/m^2^). Parameters associated with the blood test analysis, such as the total cholesterol, triglycerides, LDL-C and blood glucose, for the general participants were near the upper level limit for expected normal values. A significant association was found between gender and BMI, waist circumference, SBP and DBP, total cholesterol, LDL-C, HDL-C, triglycerides and blood glucose. Except for waist circumference, which is expected to be higher in men, most of the other health parameters were predominantly higher in men. There was also a significant association between age and the anthropometric parameters, SBP and DBP, and the blood test analysis except the HDL-C. Participants with a lower level of education, belonging to the unpaid domestic work or retired group and with the marital status of widowed show the worst health parameters associated with the anthropometric measurements, BP and blood test analysis.

### 3.3. Risk Factors for Prevalent Chronic Diseases and Multimorbidity

More than one-third of the participants were obese (35.2%), and a similar number of subjects were overweight (33.5%). Central obesity was present in 54% of the measured participants, and 43.5% had dyslipidemia. Risky alcohol consumption, tobacco smoking, low intake of fruit and vegetables, low levels of PA, and metabolic syndrome were present in 28%, 22.5%, 91.8%, 71.5%, and 21.1% of the participants, respectively. Central obesity was prevalent in women and obesity was prevalent in men; obesity and central obesity were predominant in higher ages, in subjects with lower levels of education, in the unpaid domestic workers, and in subjects who were once married. A HDL-C of risk was found to be more prevalent in men, especially among the young. Dyslipidemia was more prevalent in those with a lower level of education, among the employed and the unpaid domestic workers, and those who were married or widowed. Tobacco smoking was more prevalent among adults, employed, single or divorced. Risky alcohol consumption predominates in men and among the young. The majority of the participants were found to have a low consumption of fruit and vegetables (91.8%), as well as a low level of PA (71.5%). Participants with the lowest levels of fruit and vegetables intake were men, adults, the employed and singles. Metabolic syndrome was found to be associated with all sociodemographic characteristics and is more prevalent in men, older aged people among those with a lower level of education, in retired and domestic unpaid workers, and in the widowed and married persons. The mean number of accumulated risk factors for the general population was 4.5 ± 1.9, and it was significantly higher in men, in subjects with a lower level of education, among the group of unpaid domestic workers, retired and employed participants, and it was seen to increase with age (Table 3).

### 3.4. Characteristics of Participants with Regard to Hypertension and Diabetes

Table 4 shows the sociodemographics, lifestyle factors, anthropometrics and blood test analysis of participants with hypertension and diabetes. The prevalence of these two chronic diseases was similar among men and women, but significantly increased with age, and with lower levels of education, and among the unpaid domestic workers, the retired, and in those who once were married. Neither tobacco smoking, alcohol consumption, nor a low level of PA was associated in these participants with hypertension or diabetes. Participants with a diagnosis of diabetes had an 85.8% prevalence of low intake of fruit and vegetables, showing a small but higher consumption of fruit and vegetables than those in the general population (91.8%, see Table 3). However, hypertension and diabetes were both significantly associated with obesity, central obesity, higher BMI, higher waist circumference, higher triglycerides and blood glucose. Participants with hypertension also show higher levels of total cholesterol, and LDL-C than those participants not diagnosed with hypertension. It can also be observed in Table 4 that participants with hypertension or diabetes show similar profiles of lifestyle habits, and anthropometric measurements. Altered blood test analysis is present in both chronic health conditions but triglycerides and blood sugar levels are higher in participants with diabetes. The accumulation of several risk factors was assessed and the group of participants with hypertension or diabetes shows a significantly higher number of risk factors than participants without these chronic health conditions.

### 3.5. Characteristics of Participants with Regard to Multimorbidity

The sociodemographic, lifestyle factors, anthropometrics and blood test analysis of participants having two or more chronic health conditions are shown in Table 5. Multimorbidity was more prevalent among women, significantly increases with age, and is more prevalent in lower levels of education, among the unpaid domestic workers, the retired, and among those who once were married. About one-third of participants with multimorbidities were adults or young, and the remaining two-thirds were participants older than 64 years old. Multimorbidity was significantly associated with obesity, central obesity, higher BMI, higher waist circumferences, and higher concentrations of total blood cholesterol, LDL-C, triglycerides, and blood glucose. Both groups, with or without multimorbidities, presented a high prevalence of low levels of PA and low intake of fruit and vegetables, but the group without multimorbidity presented a lower consumption of fruit and vegetables. The accumulated number of risk factors was assessed and the group of participants with multimorbidities shows a significantly higher number of risk factors associated with chronic diseases than participants without multimorbidities.

### 3.6. Sharing of Results with Participants via Website

The total number of users accessing the www.diamantesalud.com site was 579 persons from 1 December 2014 to 30 June 2015. The webpage was visited 1174 times, and 8561 pages were navigated on the website with an average number of 7.29 pages per visit. The mean time spent consulting the website was 5 min and 46 s. The most accessed website page was inside the section of results, with 2436 visits, followed by the entrance page, and the laboratories and anthropometrics sections with 1708 and 1556 visits, respectively (Supplementary Table S1).

## 4. Discussion

This study is presenting the occurrence of chronic health conditions and multimorbidities in the central area of Argentina. The findings of the present study highlight the challenging, and health-threatening panorama marked by a high prevalence of risk factors for chronic diseases associated with obesity, central obesity, dyslipidemia, risky alcohol consumption, smoking, low levels of PA, and extremely low levels of fruit and vegetable consumption. These unhealthy lifestyle behaviors may be contributing to the development of several chronic health conditions and multimorbidities seen in this population. This research adds to the fast-growing literature about chronic diseases and multimorbidity and shows the potential application of new strategies for implementing community-based primary care.

The epidemiology of diseases in Argentina is likely influenced by its socioeconomic disparities, affecting different social groups and urban or rural areas disproportionately, and is characterized by illnesses commonly found in developed as well as in developing countries [41,42]. The basic structure of Argentinian health care is made up of three systems of health providers: (1) the public health care system, which is without cost to all subjects but is mostly used by uninsured individuals (about 34% of the population); (2) the social security system, employees and their family members (about 55% of the population); (3) and the private medical system (for about 10% of the population). The PHC is predominantly carried out for the public sector and is under the provincial Ministry of Health for each one of the 23 provinces of Argentina, not including the state of Buenos Aires. The human resources office present at the different PHC clinics throughout the country, may vary from a unique nurse technician with a weekly visit of a general practitioner, to services provided by a team which includes the daily attendance of general practitioners and a weekly visit of pediatricians, gynecologists, cardiologists, geriatologists and other medical specialists, nurses, nutritionists, physical therapists, dentistry, speech pathology, pharmacist technicians, administrative technicians, maintenance personal and community-health workers. The different provinces and even the municipalities may take autonomous decisions regarding the organization of the PHC programs and activities. A 2017 public project from the Nation Ministry of Health aims to reform the whole health system, providing universal health care coverage, with digitalized personal history and free access to essential medication for all persons, but the Argentinian health system has been marked by a paradigmatic segmented organization. This fragmentation is characterized by the different local-regional, and provincial subsystems of health care [41,42]. This structure of health care poses many challenges to the equivalent distribution of PHC services provided to people throughout the country and also affects the referral system. General practitioners may refer patients to more complex health care services but feedback to the initiating facility is not easily achieved. This may be achieved if health institutions at the operational level are supervised by central level organizations to strictly monitor referrals and ensure back referrals, and also to train staff and provide feedback [43]. The health referral system is thought to lower costs and improve equity of the health services. Suggestions to implement a functional mutual health referral system are found in a study conducted with general practitioners in Beijing [44]. There is little research on the current challenges of the Argentinian PHC system [41,42] and future studies are warranted to explore the ecology of the primary care for supporting appropriate health reforms. A recent investigation examined the ecology of medical care in Beijing. The information provided by this research is timely and may be used to improve the quality of the health care in cities around the world with similar health structures and socioeconomic environments similar to Beijing [45]. A 2016 study also shows the dynamic transitions and the important improvements that occurred recently in the Malaysian health care system [46].

Although three national representative health surveys have been conducted since 2005 in Argentina, our findings highlight important regional differences with respect to previous national reports. Obesity was found in 35.2% of the population in contrast with 18.6% for the whole province of Entre Ríos, and 20.8% nationwide in 2013. The reported prevalence of overweight in Entre Ríos and in Argentina was 37.1% while in our study it was 33.5% [47]. These differences may be associated with certain economic and sociodemographic characteristics but it is likely to be associated with limitations of data collection. While the national health survey relies on self-reported information, by contrast, our study collected data using standard body measurements and blood test analysis. Two recent research studies conducted in Argentinian cities, using anthropometric measurements, also reported higher prevalence of overweight (32.7% and 34%), and of obesity (17% and 23.5%) in the general population [48,49]. It is striking to observe that in our study, the BMI of almost three out of every four adults was above normal. Besides that, central obesity was detected in more than half of the participants. With regard to these risk factors, our results are more similar to the findings of the Hispanic Community Health Study/Study of Latinos (HCHS/SOL) living in North America [50] and to a recent study conducted in the Southern cone of Latin America [51]. Our results suggest that this population is in need of effective public health interventions to manage obesity and prevent further increases of obesity-associated diseases including diabetes, dyslipidemia, hypertension and other CVDs. New approaches for early detection and prevention and the use of some tools for implementing a more personalized medicine may be useful for community-based health interventions [52].

The assessment of sociodemographic characteristics is essential for achieving efficient and specific PHC programs. Characteristics associated with gender, age, level of education, employment status, and marital status were examined in recent PHC studies [23,24,25,26,27,28,51]. Consideration of these aspects might be especially relevant in low- and middle-income countries (LMICs), where socioeconomic and cultural inequities may predispose the conditions for the establishment of several risk factors associated with chronic diseases and multimorbidity [51,53,54,55]. In our study, certain groups of participants with common sociodemographic characteristics were identified. Altered anthropometric measurements and elevated blood biochemical risk factors for chronic diseases were predominantly found in adults, especially at older ages, in subjects with lower levels of education, in unpaid domestic workers and retirees, and among those who once were married (divorced or widowed). Nevertheless, some risk factors also predominated at younger ages such as those associated with tobacco smoking, risky alcohol consumption, low intake of fruit and vegetables, and the lower concentrations of HDL-C. Some studies have documented that unhealthy lifestyle habits at younger ages are strong predictors of chronic diseases at latter periods of life [56].

In our study, unhealthy lifestyle habits were highly reported throughout different segments of the population. More than 90% of the general population reported poor dietary habits associated with low intake of fruit and vegetables; about 70% of participants reported low levels of PA; risky alcohol consumption was found in almost 30% of participants; and smoking was found in more than 20% of participants. However, some of the unhealthy lifestyle habits were commonly reported by the younger adults, those employed, single and married participants. In contrast, some unhealthy lifestyle habits were significantly less prevalent among those participants of older ages, and those retired. The fact that the elderly show, at the present, a better lifestyle than other participants of our study, in regard to smoking, alcohol consumption and intake of fruit and vegetables, may be associated with a previous history of a medical diagnosis of chronic diseases. Indeed, a recent study from our group showed that positive lifestyle changes, especially dietary modifications, occur after the diagnosis of prostate cancer and in other types of cancer in men [57]. Similar observations have also been reported by other investigators [58,59,60]. Studies have shown that a balanced diet may decrease the risk of chronic diseases such as hypertension and diabetes. Adequate intake of fruits, green leafy vegetables, yellow/orange vegetables, and cruciferous vegetables is associated with a substantially lower risk of type 2 diabetes [61,62]. In our study, most of the participants reported a low consumption of fruit and vegetables, and these unhealthy lifestyle habits were also seen among persons with chronic diseases. Furthermore, poor lifestyle habits in persons with chronic diseases may further increase the burden of morbidity and mortality [63,64].

In our study, several risk factors for chronic diseases, MS and the accumulated number of risk factors were found to be higher in men, except for the prevalence of abdominal obesity which was higher in women. Similar results with regard to BMI were found in other studies where men showed higher prevalence of overweight and obesity [47,65]. The study of Rubinstein et al., (2015), in the Southern cone of Latin America, reported that women showed higher prevalence of obesity, abdominal obesity, risky LDL-C, and MS compared with men [51]. In our study, men showed unhealthier lifestyle habits than women, namely alcohol intake and low consumption of fruit and vegetables. Similar results were obtained in the study of Mellado-Sampedro et al., (2011), where Mexican women obtained healthier lifestyle scores than men, considering the personal health care and dietary aspects [66]. In contrast, Vidal et al., (2014), found that males in southern Chile had a better lifestyle than females when considering PA, stress management and spiritual growth [67]. In our study, there was no significant association between gender and tobacco smoking, probably because cigarette addiction is becoming more prevalent among women [68]. Although men presented more risk factors than women, multimorbidity was more prevalent in this last group. This was also observed in a Canadian study, where the threshold for the accumulated number of unhealthy lifestyle habits for presenting multimorbidity was lower in women than in men, 2 and 4, respectively [23].

When considering educational level, the group with the highest number of risk factors for chronic diseases of our study is made up of participants who had not completed basic formal education or who only finished primary school. Participants with lower levels of education presented higher values of BMI, waist circumference, SBP, DBP and altered fasting blood glucose. In addition, they presented the highest prevalence of obesity, central obesity, hypertension, dyslipidemia, MS and accumulated a number of risk factors. It is known that poor levels of education are an important cultural and sociodemographic characteristic associated with multiple chronic health conditions, including CVDs and cancer [16,23,24,69]. Stronger alliances between the educational and health sectors are needed, especially in LMICs. PHC initiatives to address this challenge should be encouraged. It is imperative to invest in public policies that allow and facilitate education in this population group [70,71,72]. To encourage health literacy among participants of our study, we used a web-based tool as well as a printed communication for devolution of results. Other investigations have successfully used similar approaches [73,74]. Similar to our study, focusing on the lifestyle and risk factors for chronic diseases, a 2016 study conducted in Utrecht found that a personalized web-based intervention has the potential to improve the lifestyle and reduce CVD risk factors among the participant subjects through changes in unhealthy behaviors [13]. The rapid pace access to mobile technologies has resulted in innumerable opportunities for preventive care through mHealth interventions. A 2016 systematic review regarding the use of eHealth/mHealth found that minority groups, such as African Americans, are among the least underrepresented in research using such technologies for health interventions [75]. This is also observed in South American countries, considering that few studies have been conducted using eHealth/mHealth approaches to improve health outcomes associated with chronic diseases [76].

Within the sociodemographic classification of labor activity, participants in the group of unpaid domestic workers (generally unemployed women and mothers who are involved in household activities), along with the retired, were those with the most risk factors for chronic diseases. These groups, who tend to spend much time at home, presented the highest prevalence for obesity, central obesity, MS and low levels of PA. Participants from these groups, who are most of the time indoors, would especially benefit from community-based PHC interventions. The interconnecting role of community health workers may be an effective way for reaching subjects to address health disparities in these groups [77].

The general prevalence of hypertension for this study was 35.9%, but increases to 41.8% if only those who had previous BP measurements and a medical diagnosis of hypertension are considered. Approximately one participant out of eight had never had a BP measurement previously. Our study also found that 16.1% of those participants who previously reported a normal BP, or the absence of hypertension, now had high BP (SBP ≥ 140 mmHg and/or DBP ≥ 90 mmHg). The general prevalence of high cholesterol was 25.8%, but increases to 43.5% if only those who had previous blood cholesterol analysis or a medical diagnosis of hypercholesterolemia are considered. Approximately one participant out of two had never had a blood cholesterol analysis. Our study also found that 34.6% of those participants who previously reported normal cholesterol levels or the absence of a diagnosis of hypercholesterolemia, now had high cholesterol (≥200 mg/dL). Similarly, the general prevalence of diabetes was 14.8%, but increases to 25.9% if only those who had previous blood glucose analysis and a medical diagnosis of diabetes are considered. Approximately one participant out of two had never had a blood glucose test. We also found that 6.5% of those participants who previously reported normal glucose levels or the absence of diabetes, now had altered blood glucose (≥110 mg/dL). Hence, the prevalence of hypertension, hypercholesterolemia and diabetes might be underestimated for this population. This may be associated with escalating unhealthy lifestyle behaviors among Argentinians [48,49]. A study assessed the accuracy of self-reported prevalence of diabetes among people who had recently checked blood glucose levels versus those who had not, and found that the prevalence was significantly lower in the latter group [78]. Our results are comparable to a 2015 study which assessed several risk factors for CVDs and found similar prevalence for obesity 35.7%, central obesity 52.9%, and hypertension 40.8%, in the Southern cone of Latin America [51].

The metabolic syndrome is a set of physical and metabolic alterations, which doubles cardiovascular risk and the risk of developing diabetes, among others chronic diseases [40,79]. The prevalence of metabolic syndrome was 21.1% if we consider the classic cut-off values, and 27.8% if we considered the most recent 2009 diagnostic criteria [80]. However, both values are below the prevalence of 37.4%, in a recent study carried out in South America [51].

When these elements are considered together, the results of our study reveal that hypertensive and diabetic participants presented a substantially higher prevalence of obesity, central obesity, waist circumference, and higher levels of cholesterol, LDL-C, triglycerides and blood glucose. The mean blood glucose level of participants with hypertension was found in pre-diabetic ranges. Our results show that participants with diabetes and hypertension shared a similar prevalence of risk factors for chronic diseases. The coexistence of several risk factors for chronic diseases may predispose to multimorbidity. Indeed, multimorbidity was identified in one-third of the participants of our study. Other investigations reported that education was inversely associated with multimorbidity, while aging was directly related [23,24,25,26,27,28]. However, a little more than one-third of participants with multimorbidities from our study were young and middle-aged adults. This has implications for Latin America countries, where the majority of the population is comprised of these groups. Our study is consistent with recent investigations that suggested that multimorbidity is not exclusively found in elderly persons [81]. Multimorbidity affects the quality of life, impairs physical and mental functioning, increases healthcare costs [15,16,20,22], and decreases the workforce of the society when the young and adults are affected. Indeed, 30.6% of those who were actively working had multimorbidity.

It is also striking to observe that all risk factors for chronic diseases related to anthropometrics, blood lipids and glucose levels, except HDL-C, were significantly associated with multimorbidity. Obesity and central obesity, for example, were found in 49.7% and 71.4%, respectively, of participants with multimorbidity. Our study corroborates the findings of other investigations, indicating that multimorbidity is highly associated with major risk factors for chronic diseases [82]. Our results also show that participants with hypertension, diabetes, and multimorbidity presented more risk factors than those without these conditions. Many modifiable chronic disease risk factors are susceptible to being managed through PHC services [83]. Therefore, to prevent further increases in multimorbidity, it is necessary to strengthen PHC services by emphasizing primary prevention, especially in highly vulnerable contexts such as in LMICs. The Argentinian health system shows an imbalance with prominence given to secondary and tertiary health care, as seen in other parts of the world, with greater expenditures in city hospitals [84,85].

A website was designed as an additional strategy for communicating with participants. The availability of a public computer center, associated with the PHC system, enabled participants to have internet access. Although the assessment of visits to this center was not possible, the computer technician expressed that several persons visited the center and were interested in getting access to the website of the project, mostly elderly participants who were not familiar with computers. Our results show that a significant number of participants have accessed the website (more than 50%). This suggests that, although computer literacy has increased dramatically in the last years, PHC projects using web-based interventions, especially in LMICs, should consider additional strategies for efficient computer use among the aged. This is one of the first studies in Argentina to use this type of community approach in a PHC program. This kind of initiative may help in raising health literacy and awareness of topics associated with health promotion and disease prevention [86]. Low-cost and effective strategies to communicate tailored health information for a large number of people at risk of chronic diseases are warranted to meet the demands of increasing occurrences of multimorbidity.

The results of our study suggest that web-based approaches are useful and have the potential to be replicated in low-resource settings of LMICs with similar PHC contexts, supporting community-based, face-to-face, primary care practices. The most visited webpage of our website was the result section containing tailored health information for the participants. Furthermore, the number of visitors also interested in searching other webpages, containing general health-promoting information about BMI, blood glucose, and dyslipidemia, suggests that displaying this kind of information to increase health literacy in this community would be beneficial. Future studies using web-based and mHealth interventions are necessary to advance interaction with people, to evaluate the changes in lifestyle habits and to optimize primary care.

Even though our research has a number of limitations, associated with observational approaches, it raises several questions and opens new avenues for future investigations, fostering research on primary care and providing new strategies for developing community-based interventions in LMICs. One of the limitations of our study was the fact that participants were not always found during community visits, and data collection was challenging. All these inconveniences delayed the time of the study and caused some missing data. Extrapolation of the data of this study to other areas of Argentina and internationally should be done with caution considering the significant socioeconomic and geographical variability and the health disparities present in low-resource settings mentioned by some of the studies cited in this paper. It may also be considered a limitation that diseases were self-reported by participants. However, recent investigations indicate that some self-reported data have a significant overlap with medical data for the most prevalent chronic diseases found in our study, such as diabetes, hypertension and hypercholesterolemia [87,88,89]. Future studies should also include other possible determinants of multimorbidity such as drug adherence, and utilization of health services as recently reported in primary care settings [90].

## 5. Conclusions

This is one of the first studies investigating the epidemiological characteristics associated with multimorbidity in a primary care context of Argentina. This research found a high prevalence of chronic disease risk factors in association with unhealthy lifestyle habits, altered anthropometrics and arterial BP measurements, and elevated blood biochemical parameters. Multimorbidity not only affected persons over 64 years of age but also youth and middle-aged adults. This may be associated with the unhealthy lifestyle habits reported by many young adults in this population. Multimorbidity was more prevalent among women, among those with lower levels of education, among those who once were married, the retired and unpaid domestic workers. The presence of multimorbidity was significantly associated with obesity, central obesity, and higher concentrations of total blood cholesterol, LDL-C, triglycerides, and blood glucose.

The feasibility of using a website for interaction within the PHC system, in a low-resource setting, was found to be a friendly environmental and cost-effective resource to support the communication of specific health-promoting information. Future studies are needed to investigate multimorbidity in Argentina. There is an urgent need to develop community-based primary health care interventions to address the risk factors associated with escalating chronic diseases and multimorbidity in South America.

## Figures and Tables

**Table 1:** Baseline characteristics of the population.

Variables	N	%
Gender (N = 1044)		
Male	365	35.0
Female	679	65.0
Age (N = 1028; mean, SD)	43	15
18–20	47	4.5
21–64	882	84.4
>64	99	9.5
Education (N = 1034)		
Primary school	423	40.5
Secondary school	369	35.3
Tertiary/University	242	23.2
Employment status (N = 1037)		
Employed	691	67.3
Unemployed	32	3.1
Students	48	4.7
Unpaid domestic work	155	15.1
Retired	101	9.8
Marital status (N = 1035)		
Single	318	30.7
Married	515	49.8
Divorced	127	12.3
Widowed	75	7.2
Hypertension * (N = 1044)	375	35.9
Diabetes * (N = 1044)	155	14.8
High Cholesterol * (N = 1044)	269	25.8
AMI or Stroke (N = 1044)	73	7.0
Asthma (N = 1044)	56	5.4
Hypothyroidism (N = 1044)	91	8.7
Celiac Disease (N = 1044)	4	0.4
Cancer (N = 1044)	15	1.4
Other chronic diseases ** (N = 1044)	146	14.0
Multimorbidities (N = 1044)	346	33.1
0	396	37.9
1	302	28.9
2	208	19.9
3	95	9.1
4	27	2.6
5	12	1.1
6	4	0.4

AMI = Acute Myocardial Infarction; * Previously diagnosed by a medical doctor; ** Arthritis, Osteoarthritis, Osteoporosis, Depression, Chronic Allergies, Chronic Pulmonary Obstructive Disease, Gastritis, Migraine, Irritable Bowel Syndrome, Epilepsy, Psychosis, Multiple Sclerosis, Psoriasis, Gout Disease, Herniated Disc, Inflammatory Vasculitis.

**Table 2:** Anthropometrics, blood pressures and blood test analysis of participants.

Characteristics	BMI (kg/m^2^) **	Waist Circumference (cm)	Systolic Blood Pressure (mmHg)	Diastolic Blood Pressure (mmHg)	Total Cholesterol (mg/dL)	LDL-C (mg/dL)	HDL-C (mg/dL)	Triglycerides (mg/dL)	Blood Glucose (mg/dL)
	Mean ± SD	Mean ± SD	Mean ± SD	Mean ± SD	Mean ± SD	Mean ± SD	Mean ± SD	Mean ± SD	Mean ± SD
General	28.71 ± 6.65	95.07 ± 17.71	124.69 ± 20.51	78.68 ± 12.02	193.05 ± 44.57	116.45 ± 36.70	49.16 ± 11.03	148.99 ± 113.65	98.20 ± 32.31
Gender	0.009 **	<0.001 **	<0.001 **	<0.001 **	NS *	0.033 *	<0.001 **	<0.001 **	<0.001 **
Male	29.25 ± 6.12	100.66 ± 16.86	129.42 ± 18.41	82.13 ± 12.07	197.41 ± 48.32	120.43 ± 40.12	44.20 ± 9.69	181 ± 144.31	105.14 ± 38.91
Female	28.42 ± 6.91	92 ± 17.43	122.11 ± 21.15	76.80 ± 11.58	190.66 ± 42.24	114.28 ± 34.54	51.87 ± 10.79	131.47 ± 88.08	94.38 ± 27.33
Age	<0.001 **	<0.001 **	<0.001 **	<0.001 **	<0.001 *	<0.001 *	NS **	<0.001 **	<0.001 **
18–20	22.86 ± 3.24	79.19 ± 9.04	109.69 ± 12.13	69.16 ± 8.07	145.16 ± 29.78	83.97 ± 25.83	45.05 ± 12.28	80.47 ± 32.95	85.74 ± 6.64
21–64	28.82 ± 6.64	94.93 ± 18.08	122.98 ± 19.32	78.71 ± 11.98	194.18 ± 44.07	117.41 ± 36.49	49.21 ± 11.13	150.35 ± 114.33	97.64 ± 31.87
>64	29.42 ± 6.72	100.52 ± 13.34	142.30 ± 21.91	81.13 ± 12.03	196.55 ± 45.72	117.01 ± 37.73	49.81 ± 10.05	157.68 ± 117.74	106.01 ± 38.22
Education	0.001 **	<0.001 **	<0.001 **	0.003 **	NS *	NS *	NS **	NS **	<0.001 **
Primary school	29.64 ± 6.91	98.63 ± 17.30	129.89 ± 22.57	80.38 ± 13.0	194.06 ± 45.5	117.32 ± 36.66	48.25 ± 10.08	151.67 ± 106.36	103.17 ± 38.05
Secondary school	28.12 ±6.40	92.88 ± 17.93	121.04 ± 18.04	77.68 ± 10.79	191.99 ± 45.56	115.37 ± 38.06	49.39 ± 11.85	148.51 ± 127.13	95.85 ± 30.63
Tertiary/University	27.83 ± 6.29	91.53 ± 16.95	120.06 ± 17.35	76.90 ± 11.38	193.08 ± 40.90	116.60 ± 34.60	50.77 ± 11.46	145.20 ± 104.55	91.53 ± 16.15
Employment status	0.001 ****	<0.001 ****	<0.001 ****	<0.001 ****	NS ***	NS ***	NS ****	0.009 ****	0.011 ****
Employed	28.49 ± 6.42	94.19 ± 18.07	123.04 ± 18.81	78.50 ± 12.03	196.10 ± 45.71	118.77 ± 38.25	49.11 ± 11.51	154.63 ± 120.40	97.69 ± 32.29
Unemployed	28.52 ± 7.67	95.97 ± 21.56	112.50 ± 11.66	73.27 ± 10.69	167.72 ± 41.44	98.77 ± 30.07	50.11 ± 11.97	106.28 ± 95.47	106.56 ± 58.93
Students	24.87 ± 5.62	85.38 ± 14.69	110.11 ± 13.47	71.78 ± 8.62	169.44 ± 39.06	97.79 ± 33.36	52.67 ± 9.01	128.67 ± 106.09	85.72 ± 8.71
Unpaid domestic work	30.57 ± 7.88	97.27 ± 17.88	125.65 ± 21.88	79.73 ± 12.41	189.92 ± 41.56	113.83 ± 32.22	49.19 ± 9.88	143.20 ± 106.34	97.79 ± 28.71
Retired	28.41 ± 5.48	99.42 ± 13.92	140.26 ± 23.02	81.82 ± 10.92	191.91 ± 41.92	114.94 ± 34.62	48.49 ± 10.48	149.48 ± 114.00	102.01 ± 29.97
Marital status	<0.001 ****	<0.001 ****	<0.001 ****	<0.001 ****	<0.001 ***	<0.001 ***	0.007 ****	0.012 ****	<0.001 ****
Single	27.14 ± 6.52	90.38 ± 19.67	118.43 ± 19.53	75.58 ± 11.26	180.16 ± 41.76	106.79 ± 33.16	48.47 ± 11.31	136.34 ±10.9.34	94.03 ± 28.60
Married	29.29 ± 6.42	96.71 ± 16.87	126.37 ± 18.92	79.97 ± 11.62	196.31 ± 42.37	118.48 ± 34.70	48.49 ± 10.96	160.94 ± 125.63	100.03 ± 34.06
Divorced	28.96 ± 6.81	96.47 ± 16.15	123.82 ± 22.12	79.28 ± 13.02	196.80 ± 47.85	119.05 ± 40.89	52.05 ± 10.98	131.40 ± 84.32	92.71 ± 17.59
Widowed	29.96 ± 7.49	98.25 ± 15.82	136.41 ± 24.36	79.99 ± 13.69	209.07 ± 53.21	131.16 ± 46.50	51.55 ± 10.03	141.75 ± 67.24	108.25 ± 44.67

BMI = Body Mass Index; LDL-C = low-density lipoprotein cholesterol; HDL-C = high-density lipoprotein cholesterol; NS = Non-significant; * *p* value for *t* test; ** *p* value for Mann–Whitney U test; *** *p* value for ANOVA; **** *p* value for Kruskal-Wallis H test.

**Table 3:** Risk factors for chronic diseases associated with the anthropometry, lifestyle and blood biochemical analysis of participants.

Characteristics	Obesity	Central Obesity	LDL-C Risk	HDL-C Risk	High Triglycerides	Dyslipidemia	Smoking	Alcohol Consumption	Low Intake of Fruits and Vegetables	Low Level of Physical Activity	Metabolic Syndrome	Total Risk Factors (N = 417)
	% (n)	% (n)	% (n)	% (n)	% (n)	% (n)	% (n)	% (n)	% (n)	% (n)	% (n) #	Mean ± SD
General	35.2 (278)	54.4 (426)	32.1 (227)	19.7 (139)	32.8 (232)	43.5 (269)	22.5 (232)	28.7 (300)	91.8 (959)	71.5 (746)	21.1 (220)	4.5 ± 1.9
Gender	0.003	0.003	0.032	<0.001	<0.001	NS	NS	<0.001	0.001	NS	0.010	<0.001 *
Male	38.1 (106)	47.3 (131)	37.2 (93)	35.6 (89)	44.8 (112)	41.8 (79)	25.6 (92)	43.0 (157)	95.6 (349)	69.8 (254)	25.5 (93)	5.1 ± 2.0
Female	33.6 (172)	58.3 (295)	29.3 (134)	10.9 (50)	26.3 (120)	44.3 (190)	20.8 (140)	21.1 (143)	89.7 (610)	72.4 (492)	18.7 (127)	4.1 ± 1.8
Age	<0.001	<0.001	0.008	0.033	0.005	NS	0.001	<0.001	<0.001	NS	<0.001	0.048 **
18–20	4.8 (1)	14.3 (3)	0 (0)	42.1 (8)	0 (0)	33.3 (3)	21.7 (10)	55.3 (26)	87.2 (41)	72.3 (34)	0 (0)	3.4 ± 1.5
21–64	35.3 (240)	53 (357)	32.6 (198)	19.6 (119)	33.2 (202)	41.8 (219)	24.1 (211)	28.1 (248)	93.4 (824)	70.9 (625)	19.8 (175)	4.5 ± 1.9
>64	41.4 (36)	76.5 (65)	36.4 (28)	15.6 (12)	39 (30)	54.9 (45)	8.1 (8)	23.2 (23)	77.8 (77)	76.8 (76)	45.5 (45)	5.0 ± 1.7
Education	0.011	<0.001	NS	NS	NS	<0.001	NS	0.014	NS	NS	<0.001	0.002 **
Primary school	42 (145)	66.3 (226)	32.2 (100)	19.3 (60)	37.3 (116)	54.3 (127)	24.7 (104)	25.4 (107)	89.6 (379)	72.6 (307)	30.3 (128)	4.9 ± 1.7
Secondary school	32.2 (88)	48.2 (131)	31.5 (79)	21.9 (55)	28.7 (72)	41 (87)	21.5 (79)	28.2 (104)	93.2 (344)	73.4 (271)	16.8 (62)	4.5 ± 2.0
Tertiary/University	25.9 (44)	40.5 (68)	32.9 (47)	16.8 (24)	30.8 (44)	31.8 (54)	20.1 (48)	36.0 (87)	93 (225)	66.1 (160)	12.4 (30)	4.1 ± 2.0
Employment status	0.027	<0.001	NS	NS	NS	0.011	0.003	<0.001	<0.001	NS	<0.001	0.011 **
Employed	33.6 (176)	47.8 (249)	35.0 (161)	21.1 (97)	33.9 (156)	45.4 (209)	25.4 (174)	33.3 (230)	94.9 (656)	70.9 (490)	17.5 (121)	4.6 ± 2.0
Unemployed	35.0 (7)	55.6 (10)	16.7 (3)	22.2 (4)	22.2 (4)	16.7 (3)	21.9 (7)	15.6 (5)	93.8 (30)	68.8 (22)	9.4 (3)	3.0 ± 1.0
Students	9.5 (2)	33.3 (7)	22.2 (4)	5.6 (1)	16.7 (3)	22.2 (4)	17.0 (8)	37.5 (18)	91.7 (44)	66.7 (32)	2.1 (1)	3.1 ± 1.2
Unpaid domestic work	46.2 (60)	71.3 (92)	25.4 (32)	17.5 (22)	29.4 (37)	34.1 (43)	22.1 (34)	12.3 (19)	87.7 (136)	76.8 (119)	32.3 (50)	4.7 ± 1.7
Retired	35.2 (31)	72.7 (64)	30.8 (24)	19.2 (15)	39.7 (31)	42.3 (33)	7.9 (8)	25.7 (26)	76.2 (77)	70.3 (71)	43.6 (44)	4.6 ± 1.6
Marital status	<0.001	<0.001	0.002	0.005	NS	<0.001	0.002	NS	0.001	NS	<0.001	NS **
Single	27.2 (58)	41.0 (86)	22.6 (42)	21.5 (40)	26.9 (50)	28.0 (52)	27.1 (85)	32.7 (104)	95.6 (304)	68.6 (218)	12.3 (39)	4.2 ± 1.7
Married	37.0 (153)	57.7 (237)	34.0 (128)	22.9 (86)	36.7 (138)	46.0 (173)	19.3 (99)	28.8 (148)	91.7 (472)	73.8 (380)	26.4 (136)	4.7 ± 2.0
Divorced	39.8 (39)	58.2 (57)	33.3 (29)	9.2 (8)	27.6 (24)	43.7 (38)	29.4 (37)	26.0 (33)	86.6 (110)	69.3 (88)	18.1 (23)	4.6 ± 2.0
Widowed	42.2 (27)	72.6 (45)	48.2 (27)	8.9 (5)	35.7 (20)	55.4 (31)	12.0 (9)	18.7 (14)	84.0 (63)	72.0 (54)	29.3 (22)	4.6 ± 1.7

* *p* value for Mann–Whitney U test; ** *p* value for Kruskal-Wallis H test.

**Table 4:** Sociodemographics, lifestyle, anthropometry and blood biochemical profile of patients with hypertension and diabetes.

Variables	Hypertensive		Diabetics	
	% (n)	*p* Value for Chi-Square Test	% (n)	*p* Value for Chi-Square Test
Gender		NS		NS
Male	32.1 (117)		14.5 (53)	
Female	38.0 (258)		15.0 (102)	
Age		<0.001		<0.001
18–20	10.6 (5)		8.5 (4)	
21–64	34.8 (307)		13.5 (119)	
>64	59.6 (59)		32.3 (32)	
Education		<0.001		<0.001
Primary school	48.6 (205)		21.1 (89)	
Secondary school	30.4 (112)		11.9 (44)	
Tertiary/University	23.6 (57)		11.7 (21)	
Employment status		<0.001		0.013
Employed	32.6 (225)		12.3 (85)	
Unemployed	21.9 (7)		18.8 (6)	
Students	18.8 (9)		6.3 (3)	
Unpaid domestic work	47.7 (74)		19.4 (30)	
Retired	56.4 (57)		27.7 (28)	
Marital status		<0.001		0.008
Single	23.9 (76)		8.2 (26)	
Married	37.4 (192)		16.0 (82)	
Divorced	49.6 (63)		18.9 (24)	
Widowed	57.3 (43)		30.7 (23)	
Smoking	22.5 (84)	NS	16.8 (26)	NS
Alcohol consumption	25.3 (95)	NS	23.2 (36)	NS
Low consumption of fruit and vegetables	90.1 (338)	NS	85.8 (133)	0.003
Low level of physical activity	71.3 (268)	NS	71.6 (111)	NS
Obesity	53.3 (163)	<0.001	52.7 (69)	<0.001
Central obesity	74.3 (223)	<0.001	70.8 (92)	0.001
BMI (kg/m^2^); mean ± SD	31.02 ± 7.23	<0.001 **	31.23 ± 6.97	<0.001 **
Waist circunference (cm); mean ± SD	100.98 ± 17.34	<0.001 **	101.15 ± 15.59	<0.001 **
Total cholesterol (mg/dL); mean ± SD	199.02 ± 43.66	0.005 *	196.63 ± 43.84	NS *
LDL-C (mg/dL); mean ± SD	120.07 ± 35.61	0.037 *	115.12 ± 35.59	NS *
HDL-C (mg/dL); mean ± SD	48.91 ± 10.74	NS **	48.45 ± 10.20	NS **
Triglycerides (mg/dL); mean ± SD	162.74 ± 112.12	<0.001 **	177.82 ± 137.19	0.002 **
Blood glucose (mg/dL); mean ± SD	105.44 ± 41.79	<0.001 **	128.31 ± 64.55	<0.001 **
Total risk factors; mean ± SD	5.10 ± 1.86	<0.001 **	5.13 ± 1.90	0.020 **

BMI = Body Mass Index; SD = Standard Deviation; * *p* value for *t* test; ** *p* value for Mann–Whitney U test.

**Table 5:** Sociodemographics, lifestyle, anthropometry and blood biochemical profile in patients with multimorbidity.

Variables	Non Multimorbidities (N = 698)	Multimorbidities (N = 346)	*p* Value for Chi-Square Test
	% (n)	% (n)	
Gender			0.019
Male	71.5 (261)	28.5 (104)	
Female	64.4 (437)	35.6 (242)	
Age			<0.001
18–20	93.4 (44)	6.4 (3)	
21–64	68.3 (602)	31.7 (279)	
>64	39.4 (39)	60.6 (60)	
Education			<0.001
Primary school	56.4 (238)	43.6 (184)	
Secondary school	73.2 (270)	26.8 (99)	
Tertiary/University	74.8 (181)	25.2 (61)	
Employment status			<0.001
Employed	69.4 (479)	30.6 (211)	
Unemployed	84.4 (27)	15.6 (5)	
Students	91.7 (44)	8.3 (4)	
Unpaid domestic work	59.4 (92)	40.6 (63)	
Retired	42.6 (43)	57.4 (58)	
Marital status			<0.001
Single	80.8 (257)	19.2 (61)	
Married	63.4 (326)	36.6 (188)	
Divorced	56.7 (72)	43.3 (55)	
Widowed	44.0 (33)	56.0 (42)	
Tobacco smoking	24.7 (170)	18.0 (62)	0.015
Alcohol consumption	31.9 (223)	22.3 (77)	0.001
Low consumption of fruit and vegetables	94.1 (657)	87.0 (301)	<0.001
Low level of physical activity	70.7 (493)	73.2 (253)	NS
Obesity	26.8 (134)	49.7 (144)	<0.001
Central obesity	44.6 (221)	71.4 (205)	<0.001
BMI (kg/m^2^); mean ± SD	27.48 ± 6.13	30.84 ± 6.98	<0.001 *
Waist circunference (cm); mean ± SD	92.22 ± 17.45	99.98 ± 17.08	<0.001 *
Total cholesterol (mg/dL); mean ± SD	187.70 ± 44.45	202.53 ± 43.27	<0.001 **
LDL-C (mg/dL); mean ± SD	113.04 ± 36.82	122.51 ± 35.77	<0.001 **
HDL-C (mg/dL); mean ± SD	49.06 ± 10.97	49.33 ± 11.17	NS *
Triglycerides (mg/dL); mean ± SD	140.42 ± 113.36	164.17 ± 112.81	0.008 *
Blood glucose (mg/dL); mean ± SD	94.0 ± 23.50	105.69 ± 42.88	<0.001 *
Total risk factors; mean ± SD	4.3 ± 1.9	5.0 ± 1.9	0.001

BMI = Body Mass Index; SD = Standard Deviation; * *p* value for Mann–Whitney U test; ** *p* value for *t* test.

## Supplementary Materials

The following are available online at www.mdpi.com/1660-4601/14/3/251/s1, Figure S1: A schematic representation of the geographical localization of Diamante city, Figure S2: The project map of the city of Diamante with the 24 studied zones delimited, and the distributions of participants with their individual code number throughout different areas of the city, Table S1: Website access statistics.
