# Supplementary material for: Risk Factors for Chronic Diseases and Multimorbidity in a Primary Care Context of Central Argentina: A Web-Based Interactive and Cross-Sectional Study
Source: Int J Environ Res Public Health. 2017 Mar 2;14(3):251. doi: 10.3390/ijerph14030251 (PMC5369087; doi:10.3390/ijerph14030251)
Supplement: Supplementary file 1 [file ijerph-14-00251-s001.pdf]

Risk Factors for Chronic Diseases and Multimorbidity in a Primary Care Context of Central Argentina: A Web-Based Interactive and Cross Sectional Study

Supplementary Table S1. Website access statistics

Pages	Number of visits	Average minutes duration
Total	8561	5:46
Results	2436	1:24
Home	1708	0:47
Laboratory and anthropometry	1556	0:29
Problems with password	479	0:46
Blood glucose	395	0:39
About the research	294	0:43
Miscellaneous	259	0:34
Cholesterol	246	0:58
Body Mass Index	217	1:00
Triglycerides	180	1:02
Contact	164	1:12

Risk Factors for Chronic Diseases and Multimorbidity in a Primary Care Context of Central Argentina: A Web-Based Interactive and Cross Sectional Study

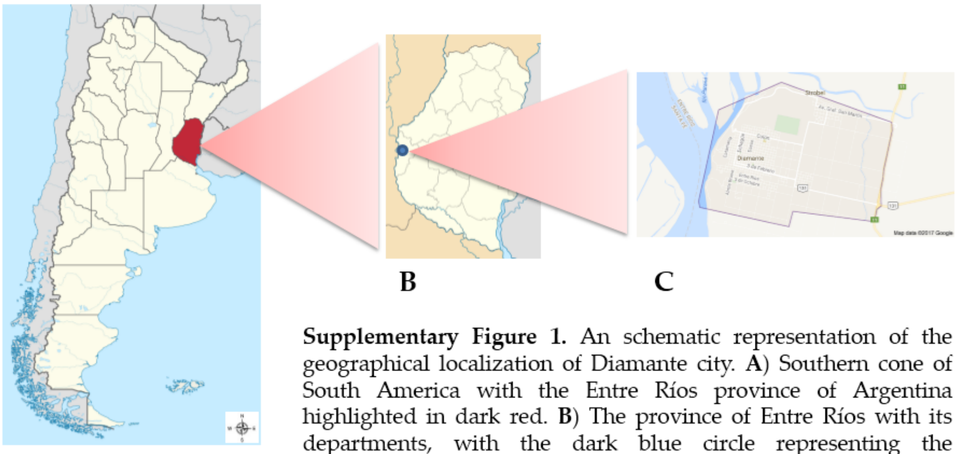

B C

A

Supplementary Figure 1. An schematic representation of the geographical localization of Diamante city. **A**) Southern cone of South America with the Entre Ríos province of Argentina highlighted in dark red. **B**) The province of Entre Ríos with its departments, with the dark blue circle representing the approximate location of the Diamante city within the department of Diamante. **C**) The city of Diamante delimited.

Figure 1A. https://commons.wikimedia.org/w/index.php?curid=17027807%3C
Figure 1B. https://es.wikipedia.org/wiki/Archivo:Argentina_Entre_R%C3%ADos_location_map.svg
Figure 1C. https://www.google.com.ar/maps/place/Diamante,+Entre+R%C3%ADos/@-32.2061915,-60.6338256,14921a,20y,43.18t/data=!3m1!1e3!4m5!3m4!1s0x95b5cda484c0de1b:0x708a22dff3fe8d55!8m2!3d-32.0697098!4d-60.6378708

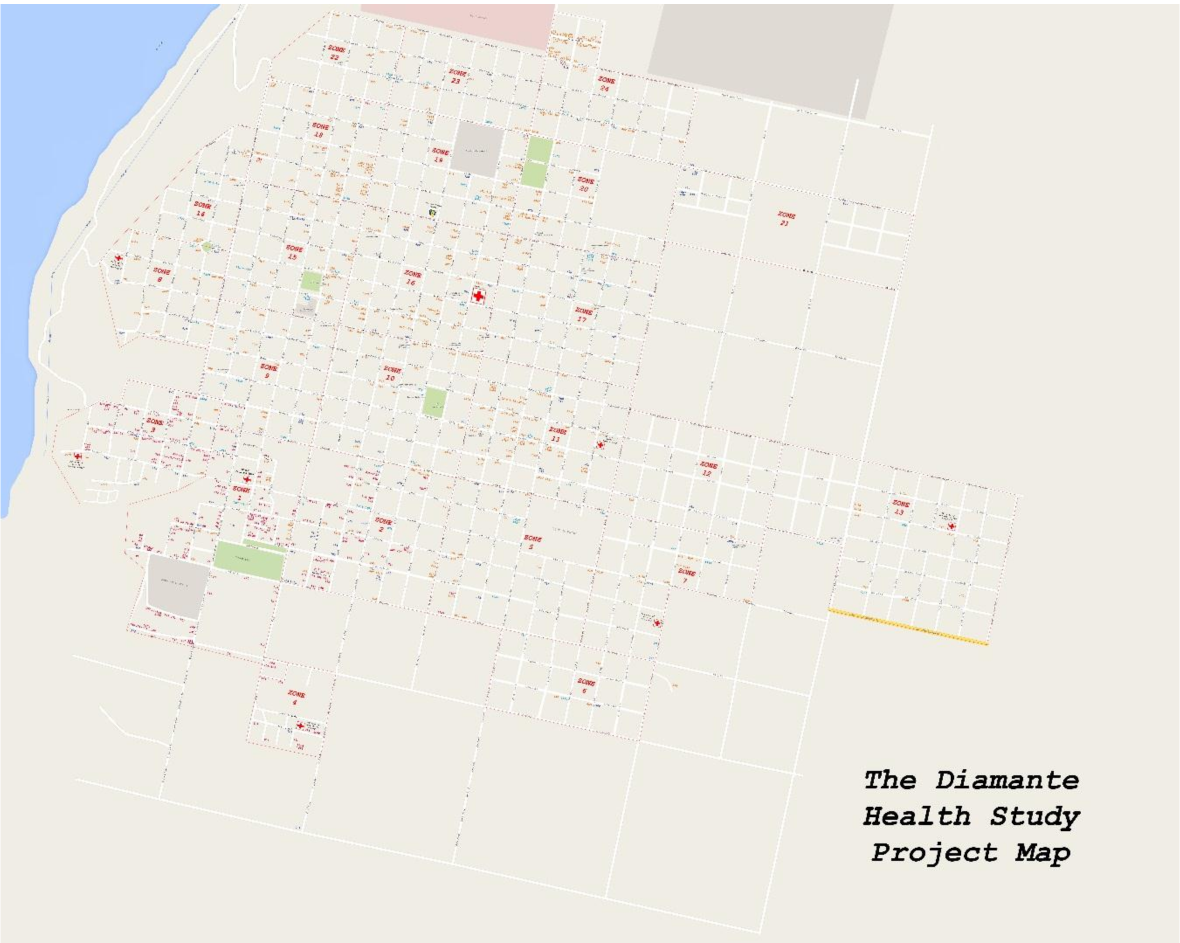

Supplementary Figure 2. The project map of the city of Diamante with the 24 studied zones delimited, and the distributions of participants with their individual code number throughout different areas of the city.
